# Long QT syndrome and torsade de pointes after anthracycline chemotherapy

**DOI:** 10.3332/ecancer.2009.147

**Published:** 2009-06-08

**Authors:** N Colombo, M Civelli, D Cardinale, G Lamantia, A Colombo, G De Giacomi, C Cipolla

**Affiliations:** Cardiology Unit, European Institute of Oncology, I.R.C.C.S, Milan, Italy

## Abstract

Anthracycline chemotherapy, which represents the treatment of choice for many hematologic and metastatic cancers, unfortunately carries with it the possibility of both early cardiotoxic phenomena, occuring during chemotherapy, and also late cardiotoxic manifestations, occuring even months or years from the completion of treatment.

The clinical manifestations of early cardiotoxicity commonly include: ventricular premature beats, supraventricular tachycardia, cardiomyopathy and sudden death.

This study confirms the necessity for close cardiac monitoring of patients undergoing anthracycline therapy. Such monitoring should not only comprise echocardiographic monitoring for left ventricular systo-diastolic dysfunction, but also electrocardiographic monitoring (QTc) in order to exclude electrophysiological changes possibly related to life threatening arrhythmias (10).

## Introduction

It is well known that the vast range of possible clinical manifestations of anthracycline cardiotoxicity includes serious arrhythmic events.

Several studies have shown how, even after a brief period of anthracycline treatment, significant increases in indexes of *ventricular recovery time* (QT, QTc, JT, JTc) may occur, which, in the absence of any other pathologic ECG findings, may represent early markers of further arrhythmias related to cardiotoxicity.

*Torsade-de-pointes* (TdP) is a life-threatening form of ventricular tachycardia and is a possible consequence of the long QT syndrome (LQTS). TdP rarely presents in the idiopathic form, but more commonly in the acquired form [1,2,3].

*Pathological QTc prolongation* is the most important sign frequently observed prior to the onset of TdP, even though there is high intra-individual and inter-individual variability.

Changes in electrolyte balance, i.e. hypokalaemia, hypocalcaemia and hypomagnesiemia, in addition to changes in hepatic and renal function, represent risk factors for TdP [4,5,6].

A wide range of chemotherapy agents have been associated with cardiotoxicity. The anthracyclines and related compounds are the most frequently implicated agents and can sometimes cause fatal cardiac arrhythmias. Individuals undergoing chemotherapy therefore require close cardiological follow-up not only during the cycles of the therapy but also at the end of it, especially those who are treated for protracted periods of time.

## Case report

A 54-year-old male with progressive micro-molecular multiple myeloma, diagnosed in August 1999 (Durie and Salmon stage IIIA: medullary infiltration 95%) with diffuse bone distribution of the disease (sternocostal, clavicular, right femoral and vertebral), underwent three cycles (1999) of vincristine, adriamycin and dexamethasone (VAD) and subsequently high-dose idarubicin (2000).

The cumulative antracycline dose received by the patient at the time of event was: adriamycin 234 mg and idarubicin 75 mg.

The patient had no clinical history of significant cardiac pathology and symptoms.

The last echocardiogram performed after completion of chemotherapy in December 2000 showed a very slight increase in the dimensions and volume of the left ventricle with normal segmentary and global kinesis (EF 55%).

The patient was admitted as an emergency on 23 January 2002 in a bad general clinical condition due to clear abdominal progression of the haemato-oncological disease. He underwent cyclic VAD chemotherapy, which resulted in a good clinical response.

The electrocardiogram (ECG) performed at admission was normal and showed a QT of 300 ms with QTc of 401 m (HR was 110 bpm).

During the night of 12 February 2002, a syncopal episode was observed.

The ECG, performed immediately after the event, while showing no significant arrhythmias, showed a significant increase in intervals (QT 450, QTc 554 ms)

The blood tests indicated a clinical picture of anaemisation (Hb: 8.9 g/dl), hypokaliemia (3.23 mEq/l), hypocalcaemia (7.1 mg/dl) not corrected by albumin level (4.0 g/dl) and normal magnesium blood levels (1.89 mg/dl).

**Figure 1:**
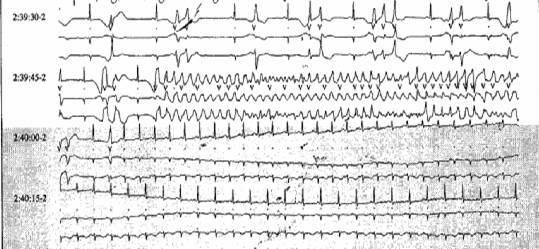
Non-sustained ventricular tachycardia of TdP-type during ECG Holter dynamic monitoring

**Figure 2:**
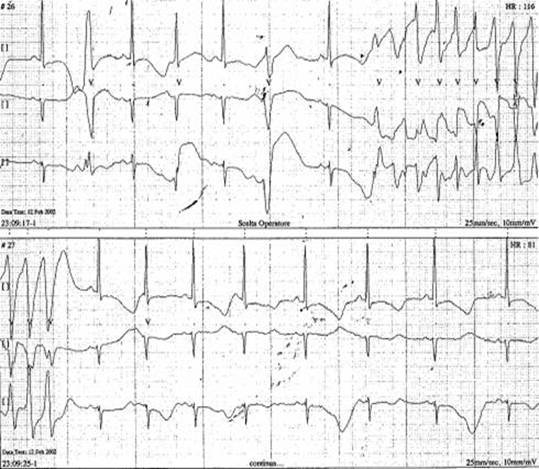
Non-sustained ventricular tachycardia of TdP-type during ECG Holter dynamic monitoring

Dynamic Holter monitoring performed the following morning, showed numerous episodes of non-*sustained* ventricular tachycardia of TdP type (Figures 1 and 2).

It was therefore decided to suspend the planned second cycle of VAD and to rapidly restore electrolytic balance, resolving the ventricular arrhythmia.

The patient was then, according to relatives, discharged.

He died at home due to the progression of the haemato-oncological disease.

## Discussion

Anthracycline chemotherapy, which represents the treatment of choice for many haematologic and metastatic cancers, unfortunately carries with it the possibility of both early cardiotoxic phenomena, occurring during chemotherapy and also late cardiotoxic manifestations, occurring even months or years from the completion of treatment.

Risk factors for the development of cardiotoxicity include: female sex, a cumulative AC dose greater than 550 mg/m^2^, co-morbidities such as arterial hypertension and pre-existing cardiac diseases, and previous mediastinal radiation therapy [7].

The clinical manifestations of early cardiotoxicity commonly include: ventricular premature beats, supraventricular tachycardia, cardiomyopathy and sudden death [8].

The late form is generally characterized by heart failure, arising from left ventricular systo-diastolic dysfunction.

Recent studies have demonstrated the mode of onset of fatal arrhythmic events, even those which occur long after the completion of anthracycline treatment and emphasize the consequent importance of long and careful follow-up in these patients [9].

Our study confirms the data in the literature, re-emphasizing the necessity for close cardiac monitoring of patients, undergoing anthracycline therapy. Such monitoring should comprise not only echocardiographic monitoring for left ventricular systo-diastolic dysfunction, but also electrocardiographic monitoring (QTc) in order to exclude electrophysiological changes possibly related to life-threatening arrhythmias [10].

At the same time, it is of prime importance to prevent or correct immediately any electrolyte imbalances, above all hypokaliemia and hypocalcaemia, which together represent a strong predisposing factor for the onset of severe and potentially fatal ventricular arrhythmias.
